# Brominated Bisindole Alkaloids from the Celtic Sea Sponge *Spongosorites calcicola*

**DOI:** 10.3390/molecules24213890

**Published:** 2019-10-29

**Authors:** Laurence K. Jennings, Neyaz M. D. Khan, Navdeep Kaur, Daniel Rodrigues, Christine Morrow, Aoife Boyd, Olivier P. Thomas

**Affiliations:** 1 Marine Biodiscovery Laboratory, School of Chemistry and Ryan Institute, National University of Ireland Galway, University Road, Galway H91 TK33, Ireland; laurence.jennings@nuigalway.ie (L.K.J.); navdeep.kaur@nuigalway.ie (N.K.); daniel.rodrigues@nuigalway.ie (D.R.); 2 National Marine Biodiscovery Laboratory of Ireland, Marine Institute, Renville West, Oranmore H91 R673, Ireland; 3 Discipline of Microbiology, School of Natural Sciences, Centre for One Health (Ryan Institute), Infection and Immunity Cluster (NCBES), National University of Ireland Galway, University Road, Galway H91 TK33, Ireland; m.khan5@nuigalway.ie (N.M.D.K.); aoife.boyd@nuigalway.ie (A.B.); 4 Department of Natural Sciences, National Museums Northern Ireland, 153 Bangor Road, Hollywood BT18 0EU, Ireland; christine.morrow@nmni.com

**Keywords:** marine natural products, sponge, cytotoxicity, bisindole alkaloid, *Spongosorites*

## Abstract

As part of an ongoing program to identify new bioactive compounds from Irish marine bioresources, we selected the subtidal sponge *Spongosorites calcicola* for chemical study, as fractions of this species displayed interesting cytotoxic bioactivities and chemical profiles. The first chemical investigation of this marine species led to the discovery of two new bisindole alkaloids of the topsentin family, together with six other known indole alkaloids. Missing the usual central core featured by the representatives of these marine natural products, the new metabolites may represent key biosynthetic intermediates for other known bisindoles. These compounds were found to exhibit weak cytotoxic activity against HeLa tumour cells, suggesting a specificity towards previously screened carcinoma and leukaemia cells.

## 1. Introduction

Marine sponges are well-recognised as producers of a diverse array of bioactive metabolites for defence, competition, and communication [1]. For this reason, these invertebrates have been the subjects of extensive chemical study over the past half century. However, there still remain extensive regions of the world where sponges have been understudied, including much of the Irish coastal waters [2]. As part of an Irish marine biodiscovery initiative supported by the Marine Institute, invertebrates present in the shallow waters of Ireland have been inventoried and then chemically and biologically screened in the search for bioactive natural products. A priority list of species placed the sponge *Spongosorites calcicola* on the top of the list due to the amount of biomass available, good chemical profiles and interesting cytotoxic activity (Table S2). While this sponge species has never been studied chemically, other species of this genus have already led to interesting bioactive metabolites.

The sponge genus *Spongosorites* has been reported to produce many brominated bisindole alkaloids belonging to four classes that differ in the linkage between the indole moieties [3,4]. These bisindoles include topsentin and spongotine derivatives with imidazole or dihydroimidazole linkages [5,6], and also, dragmacidin and hamacanthin derivatives characterised by a pyrazine or piperazine central core [7,8,9]. These bisindole alkaloids have been largely studied due to their diverse array of bioactivities [10,11,12]. Herein, we describe the isolation, structure elucidation and cytotoxic activity of two new bisindole alkaloids from the Irish sponge *S. calcicola* featuring, for the first time, an absence of cycle between the two indole rings. As such, they could represent key intermediates in the biosynthesis of known bisindole derivatives.

## 2. Results and Discussion

**Figure 1:**
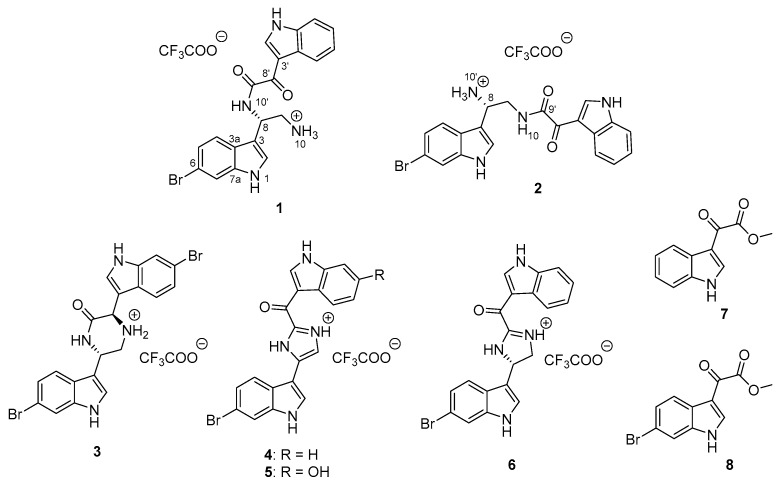
New calcicamides A and B (**1**–**2**) and known indole alkaloids, *trans*-3,4-dihydrohamacanthin A (**3**), 6-bromodeoxytopsentin (**4**), 6-bromotopsentin (**5**), spongotine A (**6**), 2-(1*H*-indol-3-yl)-2-oxoacetate methyl ester (**7**) and 2-(6-bromo-1*H*-indol-3-yl)-2-oxoacetate methyl ester (**8**) from *Spongosorites calcicola*.

The freeze-dried sponge material (5.2 g) was exhaustively extracted with a mixture of MeOH/CH_2_Cl_2_ (1:1). The resulting extract (0.97 g) was fractionated using C_18_-SPE eluting with solvent mixtures from H_2_O to MeOH to DCM. The methanol fractions, containing several brominated alkaloids as evidenced in the (+)-HRESIMS spectra, were then subjected to successive reverse-phase HPLC purifications to yield the new calcicamides A and B (**1**–**2**) as TFA salts (Figure 1). These compounds were isolated with four other known brominated bisindole alkaloids isolated from species of the genus *Spongosorites*: *trans*-3,4-dihydrohamacanthin A (**3**) [13], 6-bromodeoxytopsentin (**4**), 6-bromotopsentin (**5**) [13,14] and spongotine A (**6**) [5]. Additionally, two known monoindole derivatives related to the biosynthetic precursors were isolated from the same sample: 2-(1*H*-indol-3-yl)-2-oxoacetate methyl ester (**7**) and 2-(6-bromo-1*H*-indol-3-yl)-2-oxoacetate methyl ester (**8**).

**Figure 2:**
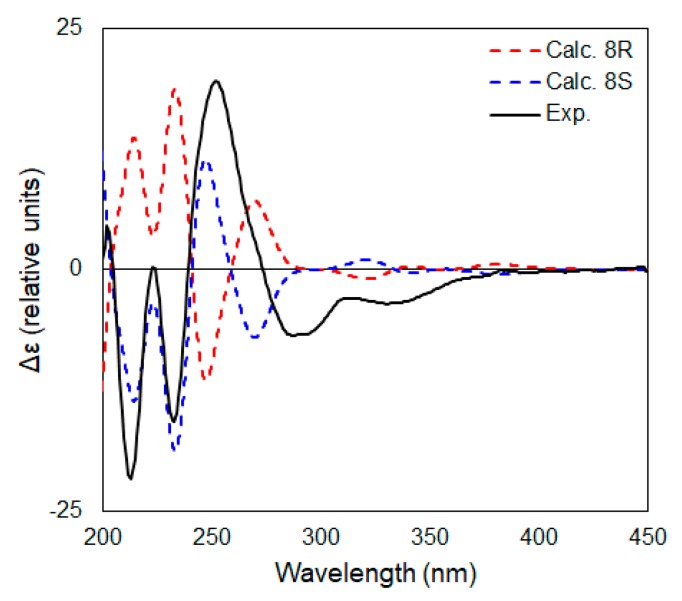
Comparison between the two TDDFT predicted electronic circular dichroism (ECD) spectra of both enantiomers of **1** and the experimental spectrum of **1**.

Compound **1** was isolated as a bright yellow amorphous solid and displayed an ion cluster in the (+)-HRESIMS spectrum for [M + H]^+^ at *m/z* 425.0614/427.0595 (1:1 ratio), consistent with the molecular formula C_20_H_18_BrN_4_O_2_. The ^1^H-NMR and ^13^C-NMR spectra performed in DMSO-*d*_6_ displayed resonances associated with one indole at δ_H_ 12.27, 8.80, 8.22, 7.53, 7.25, 7.23 and one 6-bromoindole at δ_H_ 11.29, 7.43, 7.64, 7.58, 7.18 (Table 1) [14]. The remaining signals of the ^1^H-NMR spectrum included one amide signal at δ_H_ 9.15 (d, *J* = 9.2 Hz, NH-10′), one primary amine signal at *δ*_H_ 7.98 (br s, NH_3_-10), a nitrogenated methine at δ_H_ 5.55 (td, *J* = 9.3, 4.6 Hz, H-8) and a nitrogenated methylene at δ_H_ 3.49, 3.36 (H_2_-9). A spin-coupled system between protons H-10′/H-8/H_2_-9/H_3_-10 could be assigned from the COSY spectrum. HMBC correlations from H-8 to C-2, C-3 and C-3a linked this spin coupled system to the bromoindole moiety. Finally, the ^13^C-NMR data indicated only two more resonances, associated with two carbonyls at δ_C_ 181.2 and 163.1 (C-8′ and C-9′), left to be assigned. The carbonyl chemical shifts and HMBC correlations from H-8 and H-10′ to C-9′ were in agreement with an alpha keto amide substitution on the non-brominated indole. Even though no HMBC correlations to the C-8′ carbonyl were observed, a ketone functional group at this position was supported by the presence of two absorption bands in the IR spectrum at 1672 and 1627 cm^−1^ [15]. With the 2D structure of **1** solved, the absolute configuration of the unique C-8 chiral centre was assigned through the comparison between experimental and predicted electronic circular dichroism (ECD) spectra (Figure 2). The ECD spectra of both enantiomers of **1** were calculated using Time-Dependent Density Functional Theory (TDDFT) at the B3LYP/6-311+G(2d,p)//B3LYP/6-311(2d,p) level of theory. As the experimental ECD spectrum had a close match with the calculated spectrum of the 8*S* enantiomer of **1**, we could assign the absolute configuration as *S*.

Compound **2** was isolated as a yellow amorphous solid that displayed an ion cluster in the (+)-HRESIMS for [M + H]^+^ at *m/z* 425.0608/427.0590, indicative of a structural isomer of **1**. The ^1^H-NMR and ^13^C-NMR data (Table 1) were similar to those of **1**, again revealing resonances associated with one indole (δ_H_ 12.26, 8.77, 8.22, 7.54, 7.27, 7.25) and one 6-bromoindole (δ_H_ 11.45, 7.43, 7.71, 7.63, 7.23). The differences between **1** and **2** were therefore located in the linkage between the two indole moieties. The ^13^C-NMR and IR data indicated the presence of an alpha-keto amide connected to the non-brominated indole. However, the spin-coupled system assigned through the COSY/HSQC spectra led us to identify the difference between **1** and **2** with the linkage from δ_H_ 8.95 (NH-10) to δ_H_ 3.83, 3.70 (H_2_-9) to δ_H_ 4.83 (H-8) to δ_H_ 8.33 (NH_3_-10′). The absolute configuration of the C-8 stereocenter was again determined by ECD analysis. The theoretical ECD spectra of the enantiomers of **2** were calculated using the same methods and basis set/functional combination as used for **1**, allowing the assignment of the same 8*S* configuration for calcicamide B (Figure S15).

**Scheme 1:**
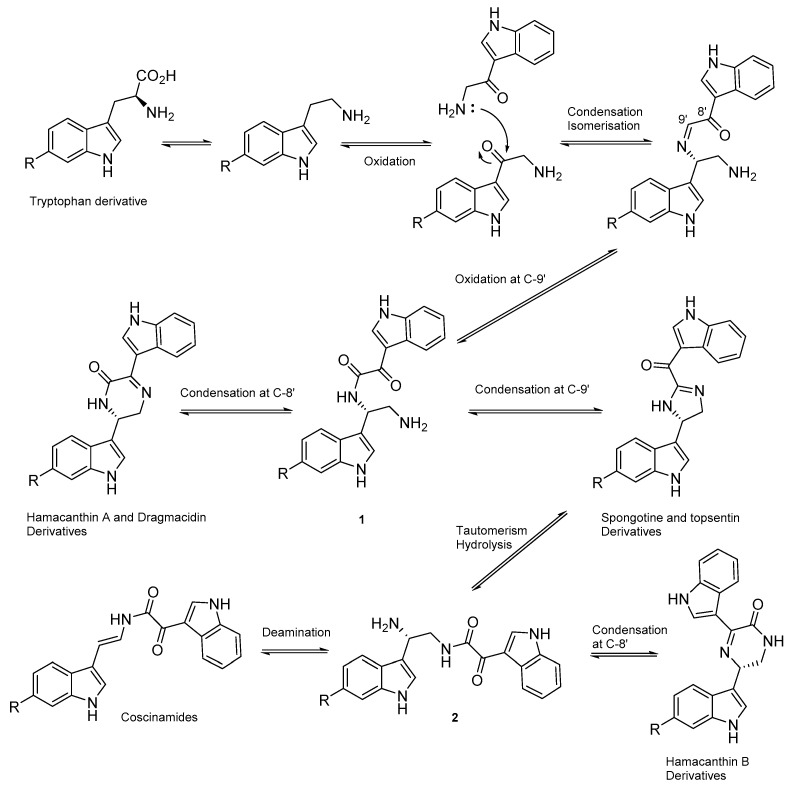
Biosynthetic hypothesis for the calcicamide, hamacanthin, coscinamides, and topsentin derivatives.

Calcicamides (**1**–**2**) differ from other bisindole compounds due to the lack of a central heterocyclic ring, which is instead opened in **1** and **2**. As the tryptophan origin of bisindole alkaloids cannot be contested, we hypothesize that the nucleophilicity of one primary amine should enable the connection between two 8-ketotryptamine derivative units after a first condensation (Scheme 1) [3]. Hydration/oxidation of the intermediate would lead to compound **1**. Oxidation of the 8-ketotryptamine derivatives at C-9 prior to isomerisation would lead to the monoindole derivatives **7** and **8**. The piperazine central core of hamacanthins and dragmacidins would subsequently be formed when the second condensation occurs at C-8′ of compound **1** or **2**. To assess if calcicamides are natural products or hydrolysed artefacts produced during the isolation process, the extracts and fractions were analysed by LC-MS/MS prior to acidic HPLC isolation. We identified two peaks in the chromatogram with a molecular ion at *m/z* 425/427 (1:1) coeluting with the two isomers of calcicamides. This result came as a proof of their natural origin. Additionally, coscinamides isolated from the marine sponge *Coscinoderma* sp. are another family of bisindole alkaloids that lack a central ring and instead contain a linear chain with an alpha keto enamide [15]. We hypothesise that the coscinamides may also be biosynthetically related to calcicamide B (**2**) following a deamination at C-8.

Members of the bisindole alkaloid family have been previously reported with a large array of bioactivities including moderately potent antiviral, antitumoral, antibacterial, and antifungal activities [5,10,16]. The six bisindole alkaloids were screened for their antitumoral activity against a HeLa cell line. Compounds **2** and **4**–**6** exhibited weak activity against these cells with IC_50_s between 100 and 200 µM (Table S1). Compounds **1** and **3** did not induce detectable cell lysis at the highest tested concentrations (185 and 166 µM respectively). Most cell death occurred within the first 6 h of incubation. Cell lysis increased between 6 and 24 h incubation, corresponding to an increase in the cytotoxicity with longer exposure. Compounds **3**–**6** have previously been shown to have moderate cytotoxicity against adenocarcinoma (AGS) and lymphocytic leukaemia (L1210) cells. This previous study noted that the cytotoxicity of *Spongosorites* bisindoles is cell-line dependent with only weak activity reported against other cell lines screened [10]. The inability of these compounds to display comparable activity against HeLa cells again suggests that these compounds may be more specific against carcinoma and leukaemia cell lines. Therefore, based on these results we will continue a deeper evaluation of the cytotoxicity of *Spongosorites* bisindoles against carcinoma and leukaemia cell lines.

## 3. Materials and Methods

### 3.1. General Experimental Procedures

Optical rotations were recorded on a Unipol L1000 polarimeter at the sodium D-line (589.3 nm) with a 5 cm cell at 20 °C (Schmidt+Haensch, Berlin, Germany). UV and ECD were recorded on a Chirascan V100 with a 1.0 cm quartz cuvette (Applied Photophysics, Leatherhead, UK). IR data were recorded on a PerkinElmer spectrum 100 FT-IR spectrometer (PerkinElmer, Waltham, MA, USA). NMR experiments were performed on a 500 MHz Varian Inova spectrometer (Agilent, Santa Clara, CA, USA). Chemical shifts (δ in ppm) were referenced to the carbon (δ_C_ 39.52) and proton (δ_H_ 2.50) signals of DMSO-*d*_6_. HRESIMS were obtained using an Agilent 6540 Q-Tof mass spectrometer equipped with an Agilent 1290 UPLC and autosampler (Agilent). Preparative and semipreparative HPLC was carried out on a Jasco LC-2000 series equipped with a coupled UV detector. Analytical HPLC was carried out on an Agilent 1260 HPLC system equipped with a DAD detector coupled with an Agilent 385-ELSD. All solvents used for extraction and separation were HPLC grade, and H_2_O was Milli-Q (Millipore Ireland B.V., Carrigtwohill, County Cork, Ireland) filtered.

### 3.2. Biological Material

*Spongosorites calcicola* was collected at Rathlin Island (Co. Antrim, Northern Ireland) at 18 m depth by SCUBA. The sponge was taxonomically identified by Christine Morrow through morphological and spicule analysis. A voucher specimen of this sample “BDV10015” is kept at the National Marine Biodiscovery Laboratory (Marine Institute, Oranmore, Co. Galway, Ireland).

### 3.3. Extraction and Isolation

The freeze-dried sponge material (5.20 g) was extracted with a solvent mixture of 1:1 (*v*/*v*) MeOH/CH_2_Cl_2_ under sonication to yield an extract (970 mg). The extract was then fractionated using SPE on C_18_-bonded silica gel, eluting with varying solvent mixtures to yield five fractions: 100% H_2_O, 50% H_2_O/50% MeOH (331.9 mg), 25% H_2_O/75% MeOH (115.7 mg), 100% MeOH (67.8 mg), 50% MeOH/50% CH_2_Cl_2_ (81.5 mg). The 75% and 100% MeOH fractions with similar profiles were then combined for HPLC purification. The resulting fraction was separated using preparative HPLC on a phenyl-hexyl column (Waters, Xselect CSH phenyl-hexyl 130 Å, 5 µm, 19 × 250 mm). The column was first eluted with 90% H_2_O (0.1% TFA)/10% MeOH (0.1% TFA) for 5 min, followed by a linear gradient to 100% MeOH (0.1% TFA) over 20 min, and the column was then further eluted with the final conditions for 7 min, all at a flow rate of 10 mL/min. This resulted in the collection of six peaks from the UV chromatogram; these peaks were dried and re-purified using semi-preparative and analytical HPLC.

Peak 2 (11.63 mg) was re-purified on a C_18_ analytical column (Macherey-Nagel Nucleodur HTec C_18_, 5 µm, 4.6 × 250 mm) eluting first with 74% H_2_O (0.1% TFA)/26% MeOH (0.1% TFA) for 5 min, followed by a linear gradient to 50% H_2_O (0.1% TFA)/50% MeOH (0.1% TFA) over 20 min, another gradient to 100% MeOH (0.1% TFA) over 10 min was performed and the column was then further eluted with the final conditions for 7 min, all at a flow rate of 1.0 mL/min. This yielded the new compound calcicamide A (**1**, 6.24 mg, 0.12% dry wt.) and the known compound *trans*-3,4-dihydrohamacanthin A (**3**, 3.11 mg, 6.0 × 10^−2^% dry wt.).

Peaks 3 and 4 were combined (82.74 mg) and re-purified on a C_18_ semiprep column (Waters symmetry C18 prep, 7 µm, 7.8 × 250 mm) eluting with isocratic conditions of 62% H_2_O (0.1% TFA)/38% ACN (0.1% TFA) for 25 min at a flow rate of 4.0 mL/min. This yielded the known compound spongotine A (**6**, 39.03 mg, 0.75% dry wt.), the new compound calcicamide B (**2**, 7.8 mg, 0.15% dry wt.), and the known compounds bromotopsentin (**5**, 42.12 mg, 0.81% dry wt.) and 2-(1H-indol-3-yl)-2-oxoacetate methyl ester (**7**, 1.4 mg, 2.6 × 10^−2^% dry wt.).

Peak 5 (39.24 mg) was re-purified on a C_18_ analytical column (Macherey-Nagel Nucleodur HTec C_18_, 5 µm, 4.6 × 250 mm) eluting first with 90% H_2_O (0.1% TFA)/10% MeOH (0.1% TFA) for 5 min, followed by a linear gradient to 100% MeOH (0.1% TFA) over 20 min, and the column was further eluted with the final conditions for 10 min, all at a flow rate of 1.2 mL/min. This yielded the known compound bromodeoxytopsentin (**4**, 37.96 mg, 0.73% dry wt.). Peak 6 (24.02 mg) was re-purified with the same column and conditions used for peak 5. This yielded the known compound 2-(6-bromo-1H-indol-3-yl)-2-oxoacetate methyl ester (**8**, 1.0 mg, 1.9 × 10^−2^% dry wt.).

Calcicamide A (**1**): Bright yellow, amorphous solid; ${\left[\alpha \right]}_{D}^{20}$ + 0.3 (*c* 0.1, CH_3_CN); UV/Vis (*c* 1 × 10^−4^ M, CH_3_CN) λ_max_ (log ε) 329 (3.50), 293 (3.42), 280 (3.63), 245 (3.63), 213 (4.14) nm; ECD (*c* 1 × 10^−4^ M, CH_3_CN) λ_max_ (Δε) 331 (−0.2), 287 (−0.37), 252 (+1.06), 233 (−0.86), 213 (−1.18) nm; ^1^H-NMR (DMSO-*d*_6_, 500 MHz) and ^13^C-NMR data see Table 1; HRESIMS (+) *m/z* 425.0614 [M + H]^+^ (calcd. for C_20_H_18_^79^BrN_4_O_2_^+^, 425.0608, Δ + 1.4 ppm).

Calcicamide B (**2**): Bright yellow, amorphous solid; ${\left[\alpha \right]}_{D}^{20}$ + 0.2 (*c* 0.1, CH_3_CN); UV/Vis (*c* 5 × 10^−5^ M, CH_3_CN) λ_max_ (log ε) 314 (3.23), 239 (3.40), 210 (3.87) nm; ECD (*c* 5 × 10^−5^ M, CH_3_CN) λ_max_ (Δε) 335 (−0.93), 272 (−1.48), 244 (−1.39), 231 (+0.63), 218 (−0.13) nm; ^1^H-NMR (DMSO-*d*_6_, 500 MHz) and ^13^C-NMR data see Table 1; HRESIMS (+) *m/z* 425.0608 [M + H]^+^ (calcd. for C_20_H_18_^79^BrN_4_O_2_^+^, 425.0608, Δ + 0.0 ppm).

*trans-3,4-Dihydrohamacanthin A* (**3**) [13]: Yellow, amorphous solid; ^1^H-NMR (DMSO-*d*_6_, 500 MHz) δ 11.54 (1H, s), 11.48 (1H, s), 10.06 (1H, brd, *J* = 9.7 Hz), 9.83 (1H, brq, *J* = 9.4 Hz), 8.72 (1H, d, *J* = 2.6 Hz), 7.82 (1H, d, *J* = 8.6 Hz), 7.67 (1H, s), 7.63 (1H, d, *J* = 1.7 Hz), 7.62 (1H, d, *J* = 1.7 Hz), 7.54 (1H, s), 7.53 (1H, d, *J* = 8.6 Hz), 7.27 (1H, dd, *J* = 8.6, 1.7 Hz), 7.23 (1H, dd, *J* = 8.6, 1.7 Hz), 5.71 (1H, m), 5.37 (1H, m), 4.09 (1H, m), 3.64 (1H, m) and ^1^H-NMR (MeOH-*d*_4_, 500 MHz) δ 7.77 (1H, d, *J* = 8.6 Hz), 7.61 (1H, d, *J* = 1.7 Hz), 7.61 (1H, d, *J* = 1.7 Hz), 7.58 (1H, s), 7.56 (1H, d, *J* = 8.6 Hz), 7.52 (1H, s), 7.30 (1H, dd, *J* = 8.6, 1.7 Hz), 7.26 (1H, dd, *J* = 8.6, 1.7 Hz), 5.80 (1H, s), 5.37 (1H, m), 4.09 (1H, m), 3.64 (1H, m); HRESIMS (+) *m/z* 486.9769/488.9747/490.9726 (1:2:1) [M + H]^+^ (calcd. for C_20_H_17_^79^Br_2_N_4_O^+^, 486.9764, Δ + 1.0 ppm).

*Bromodeoxytopsentin* (**4**) [13,14]: Yellow, amorphous solid; ^1^H-NMR (DMSO-*d*_6_, 500 MHz) δ 12.13 (1H, brs), 11.51 (1H, brs), 9.20 (1H, s), 8.36 (1H, brd, *J* = 7.3 Hz), 7.98 (1H, d, *J* = 8.4 Hz), 7.94 (1H, s), 7.68 (1H, s), 7.64 (1H, s), 7.54 (1H, brd, *J* = 7.0 Hz), 7.24 (3H, m) and ^13^C-NMR data (DMSO-*d*_6_, 125 MHz) δ 175.9, 145.2, 137.3, 136.9, 136.2, 126.7, 124.3 (br), 123.8, 122.9, 122.4, 121.9, 121.7, 121.6, 114.4, 114.3, 113.7, 112.3 (NOTE: some carbons could not be observed); HRESIMS (+) *m/z* 405.0352/407.0330 (1:1) [M + H]^+^ (calcd. for C_20_H_14_^79^BrN_4_O^+^, 405.0346, Δ + 1.5 ppm).

*Bromotopsentin* (**5**) [13,14]: Yellow, amorphous solid; ^1^H-NMR (DMSO-*d*_6_, 500 MHz) δ 11.83 (1H, d, *J* = 2.4 Hz), 11.53 (1H, brs), 8.99 (1H, d, *J* = 2.4 Hz), 8.14 (1H, d, *J* = 8.6 Hz), 8.00 (1H, d, *J* = 8.5 Hz), 7.99 (1H, d, *J* = 2.2 Hz), 7.73 (1H, s), 7.66 (1H, d, *J* = 1.7 Hz), 7.25 (1H, dd, *J* = 8.5, 1.7 Hz), 6.91 (1H, d, *J* = 2.0 Hz), 6.77 (1H, dd, *J* = 8.6, 2.0 Hz) and ^13^C-NMR data (DMSO-*d*_6_, 125 MHz) δ 175.2, 154.4, 144.8, 137.6, 137.4, 136.0, 133.5 (br), 124.6, 123.7, 122.5, 122.1, 121.6, 119.5, 119.1 (br), 114.5, 114.4, 113.9, 112.2, 107.5 (br), 97.6; HRESIMS (+) *m/z* 421.0302/423.0280 (1:1) [M + H]^+^ (calcd. for C_20_H_14_^79^BrN_4_O_2_^+^, 421.0295, Δ + 1.7 ppm).

*Spongotine A* (**6**) [5]: Yellow, amorphous solid; [α]_20_^D^ –9.2 (c 1.0, ACN); ^1^H-NMR (DMSO-*d*_6_, 500 MHz) δ 12.86 (1H, s), 11.50 (1H, s), 11.34 (1H, brs), 11.14 (1H, s), 8.57 (1H, d, *J* = 3.5 Hz), 8.18 (1H, dd, *J* = 7.0, 2.1 Hz), 7.67 (1H, d, *J* = 1.7 Hz), 7.66 (1H, d, *J* = 2.5 Hz), 7.62 (1H, dd, *J* = 7.0, 1.8 Hz), 7.56 (1H, d, *J* = 8.5 Hz), 7.38 (1H, td, *J* = 7.0, 2.1 Hz), 7.36 (1H, td, *J* = 7.0, 1.8 Hz), 7.25 (1H, dd, *J* = 8.5, 1.7 Hz), 5.87 (1H, dd, *J* = 12.2, 9.3 Hz), 4.52 (1H, t, *J* = 12.0 Hz), 4.08 (1H, dd, *J* = 11.6, 9.3 Hz) and ^13^C-NMR data (DMSO-*d*_6_, 125 MHz) δ 173.1, 161.7, 140.1, 137.6, 137.3, 125.9, 125.0, 124.7, 124.0, 123.7, 122.3, 121.2, 120.1, 114.8, 114.7, 113.4, 113.3, 112.6, 54.4, 51.3; HRESIMS (+) *m/z* 407.0512/409.0489 (1:1) [M + H]^+^ (calcd. for C_20_H_16_^79^BrN_4_O^+^, 407.0502, Δ + 2.5 ppm).

*2-(1H-Indol-3-yl)-2-oxoacetate methyl ester* (**7**) [3]: Yellow, amorphous solid; ^1^H-NMR (CD_3_OD-*d*_4_, 500 MHz) δ 12.47 (1H, s), 8.47 (1H, d, *J* = 3.3 Hz,), 8.08 (1H, d, *J* = 8.5 Hz,), 7.73 (1H, d, *J* = 1.7 Hz,), 7.41 (1H, dd, *J* = 8.5, 1.8 Hz,), 3.88 (3H, s); HRESIMS (+) *m/z* 204.0665 [M + H]^+^ (calcd. for C_11_H_10_NO_3_^+^, 204.0655, Δ + 4.9 ppm).

*2-(6-Bromo-1H-indol-3-yl)-2-oxoacetate methyl ester* (**8**) [3]: Yellow, amorphous solid; ^1^H-NMR (DMSO-*d*_6_, 500 MHz) δ 12.47 (1H, s), 8.47 (1H, d, *J* = 3.3 Hz,), 8.08 (1H, d, *J* = 8.5 Hz,), 7.73 (1H, d, *J* = 1.7 Hz,), 7.41 (1H, dd, *J* = 8.5, 1.8 Hz,), 3.88 (3H, s); HRESIMS (+) *m/z* 281.9763/283.9745 (1:1) [M + H]^+^ (calcd. for C_11_H_9_^79^BrNO_3_^+^, 281.9760, Δ + 1.1 ppm).

### 3.4. Computational Methods

Conformational analyses of each compound were performed with Schrodinger MacroModel [17]. These conformers were then optimized using DFT, at the B3LYP/6-311+G(2d,p) level in Gaussian 16. At the same time, the zero-point energy, electronic transition, and rational strength of all conformers were calculated. Following this the ECD spectra for each conformer were calculated in Gaussian 16 at the B3LYP/6-311G(2d,p) level [18]. All DFT calculations were performed using a polarisable continuum model [19]. The final ECD spectra were produced using the freely available software SpecDis 1.7 (version 1.71, SpecDis, Berlin, Germany) and corrected with the experimental UV spectra [20].

### 3.5. Biological Screening

HeLa cells were cultured in DMEM complete (Dulbecco’s Minimal Eagles Medium-low glucose and without phenol red containing 10% (*v*/*v*) foetal bovine serum (FBS), non-essential amino acids, 20 mmol L-glutamine, 100 μg·mL^−1^ penicillin and 100 μg·mL^−1^ streptomycin) at 37 °C, 5% CO_2_. For co-incubation experiments, cells were seeded at 20,000 cells per mL DMEM complete per well in 48-well tissue culture plates. After 16 h, monolayers were washed with PBS and 500 μL DMEM was added per well with the sponge fractions (100 μg/mL) or isolated molecules (12–200 μM). Non-treated cells acted as the 0% lysis control and cells treated with 0.8% Triton X-100 were the 100% lysis control. After 6 and 24 h, cytotoxicity was determined using the Promega CytoTox 96 Non-Radioactive Cytotoxicity Assay kit, according to the manufacturer’s directions, to measure lactate dehydrogenase released from lysed cells. Data are presented as means ± SD of three independent experiments performed.

## 4. Conclusions

The first chemical investigation of the sponge *Spongosorites calcicola* collected in the Celtic Sea led to the isolation of two new bisindole alkaloids that are likely important biosynthetic intermediates in the metabolic pathway to known analogues. Bisindole alkaloids represent an important class of sponge natural products for pharmaceutical applications, and our study highlighted some selectivity for cytotoxicity.

## Figures and Tables

**Table 1:** NMR data for **1** and **2** in DMSO-*d*_6_ (^1^H-NMR at 500 MHz and ^13^C-NMR at 125 MHz).

Pos.	1			2		
	δ_C_, type	δ_H_ (*J*·in Hz)	HMBC	δ_C_, type	δ_H_ (*J*·in·Hz)	HMBC
1		11.29, d (2.3)	2, 3, 3a, 7a		11.45, d (2.2)	2, 3, 3a, 7a
2	124.6, CH	7.43, d (2.3)	3, 3a, 7a	125.3, CH	7.58, d (2.6)	3, 3a, 7a
3	112.6, C			109.6, C		
3a	124.5, C			124.7, C		
4	120.3, CH	7.64, d (8.7)	3, 6, 7a	120.3, CH	7.71, d (8.5)	3, 6, 7a
5	121.8, CH	7.18, dd (8.7, 1.9)	3a, 6, 7	122.2, CH	7.23, dd (8.5, 1.7)	3a, 6, 7
6	114.2, C			114.5, C		
7	114.3, CH	7.58, d (1.9)	3a, 5, 6	114.6, CH	7.63, d (1.7)	3a, 5, 6
7a	137.0, C			136.8, C		
8	42.7, CH	5.55, td (9.3, 4.6)	2, 3, 3a, 9′	46.9, CH	4.83, q (6.2)	2, 3, 3a, 9
9	41.1, CH_2_	3.49, m 3.36, under H_2_O		42.1, CH_2_	3.83, dt (13.8, 7.2) 3.70, dt (13.8, 5.7)	3, 8, 9′
10-NH		7.98, br s			8.95, t (6.1)	9, 9′
1′		12.27, d (3.2)	2′, 3′		12.26, d (3.0)	2′, 3′,3′a,7′a
2′	138.6, CH	8.80, d (3.2)	3′, 3′a, 7′a	138.7, CH	8.77, d (3.3)	3′, 3′a, 7′a
3′	112.1, C			112.1, C		
3′a	126.3, C			126.3, C		
4′	121.2, CH	8.22, dd (7.2, 2.1)	6′, 7′a	121.3, CH	8.22, dd (6.8, 2.3)	6′, 7′a
5′	122.6, CH	7.23, td (7.2, 2.0)	3′a, 7′	122.7, CH	7.25, td (6.9, 2.2)	3′a, 7′
6′	123.5, CH	7.25, td (7.2, 2.0)	4′, 7′a	123.6, CH	7.27, td (6.9, 2.3)	4′, 7′a
7′	112.6, CH	7.53, dd (7.2, 2.0)	3′a, 5′	112.7, CH	7.54, dd (7.0, 2.2)	3′a, 5′
7′a	136.2, C			136.2, C		
8′	181.2, C			180.9, C		
9′	163.1, C			163.7, C		
10′-NH		9.15, d (9.2)	8, 9′		8.33, br d (4.3)	3

## Supplementary Materials

Electronic supplementary information including MS, NMR, IR and ECD spectra of new compounds is available online at https://www.mdpi.com/1420-3049/24/21/3890/s1.
