# Supplementary material for: Brominated Bisindole Alkaloids from the Celtic Sea Sponge Spongosorites calcicola
Source: Molecules. 2019 Oct 29;24(21):3890. doi: 10.3390/molecules24213890 (PMC6864463; doi:10.3390/molecules24213890)
Supplement: Supplementary file 1 [file molecules-24-03890-s001.pdf]

molecules

Supplementary data

Brominated Bisindole Alkaloids from the Celtic Seas Sponge *Spongosorites calcicola*

Laurence K. Jennings[1,2], Neyaz M. D. Khan[3], Navdeep Kaur[1,2], Daniel Rodrigues[1,2], Christine Morrow[4], Aoife Boyd[3] and Olivier P. Thomas[1,*]

1. School of Chemistry, Ryan Institute, National University of Ireland Galway, University Road, Galway H91 TK33. Ireland. laurence.jennings@nuigalway.ie, navdeep.kaur@nuigalway.ie, daniel.rodrigues@nuigalway.ie, olivier.thomas@nuigalway.ie.
2. National Marine Biodiscovery Laboratory of Ireland, Marine Institute, Renville West, Oranmore H91 R673. Ireland. laurence.jennings@marine.ie, navdeep.kaur@marine.ie, daniel.rodrigues@marine.ie
3. Discipline of Microbiology (School of Natural Sciences), Centre for One Health (Ryan Institute), Infection and Immunity Cluster (NCBES), National University of Ireland Galway, University Road, Galway, H91 TK33, Ireland. m.khan5@nuigalway.ie, aoife.boyd@nuigalway.ie.
4. Department of Natural Sciences, National Museums Northern Ireland, 153 Bangor Road, Holywood BT18 0EU, Northern Ireland. christine.morrow@nmni.com.

* Correspondence: olivier.thomas@nuigalway.ie; Tel.: +353-(0)-8-9455-6289

P2 **Figure S1.** ^{1}H NMR spectrum of **1** at 500 MHz in DMSO-*d*$_6$.
P2 **Figure S2.** ^{13}C NMR spectrum of **1** at 125 MHz in DMSO- *d*$_6$.
P3 **Figure S3.** COSY NMR spectrum of **1** at 500 MHz in DMSO- *d*$_6$.
P3 **Figure S4.** HSQC NMR spectrum of **1** at 500 MHz in DMSO- *d*$_6$.
P4 **Figure S5.** HMBC NMR spectrum of **1** at 600 MHz in DMSO- *d*$_6$.
P4 **Figure S6.** HMBC NMR spectrum of **1** at 600 MHz in DMSO- *d*$_6$.
P5 **Figure S7.** IR spectrum of **1**.
P5 **Figure S8.** (+)ESIHRMS spectrum of **1**
P6 **Figure S9.** ^{1}H NMR spectrum of **2** at 500 MHz in DMSO- *d*$_6$
P6 **Figure S10.** ^{13}C NMR spectrum of **2** at 125 MHz in DMSO- *d*$_6$.
P7 **Figure S11.** COSY NMR spectrum of **2** at 500 MHz in DMSO- *d*$_6$.
P7 **Figure S12.** HSQC NMR spectrum of **2** at 500 MHz in DMSO- *d*$_6$.
P8 **Figure S13.** HMBC NMR spectrum of **2** at 600 MHz in DMSO- *d*$_6$.
P8 **Figure S14.** HMBC NMR spectrum of **2** at 600 MHz in DMSO- *d*$_6$.
P9 **Figure S15.** Experimental and calculated ECD spectrum of **2**.
P9 **Figure S16.** (+)ESIHRMS spectrum of **2**
P10 **Table S1.** Cytotoxicity (μM) of compounds **1-6** against HeLa cells measured after 6 and 24 h incubation.
P10 **Table S2.** Cytotoxicity (μg/mL) of fractionated sponge extracts against HeLa cells measured after 6 and 24 h incubation.

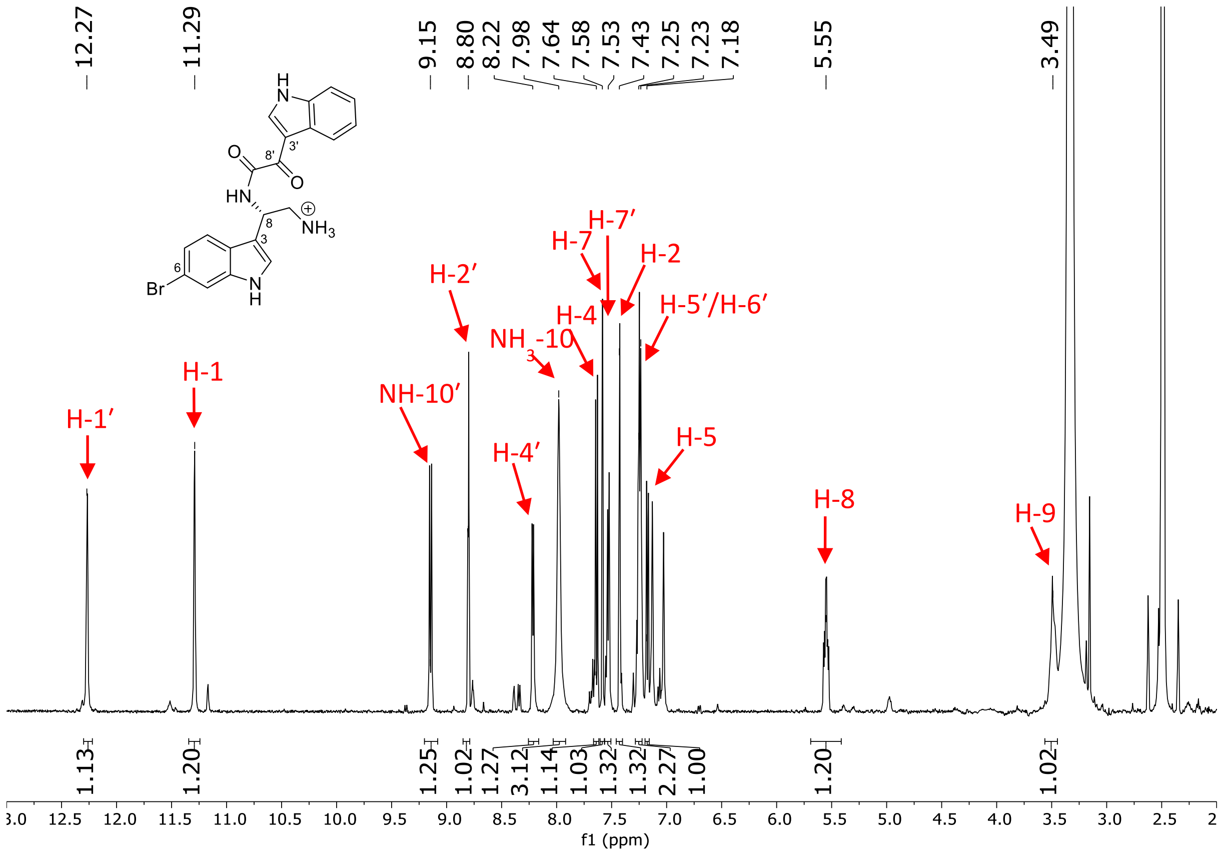

Figure S1. ^{1}H NMR spectrum of **1** at 500 MHz in DMSO-*d*6.

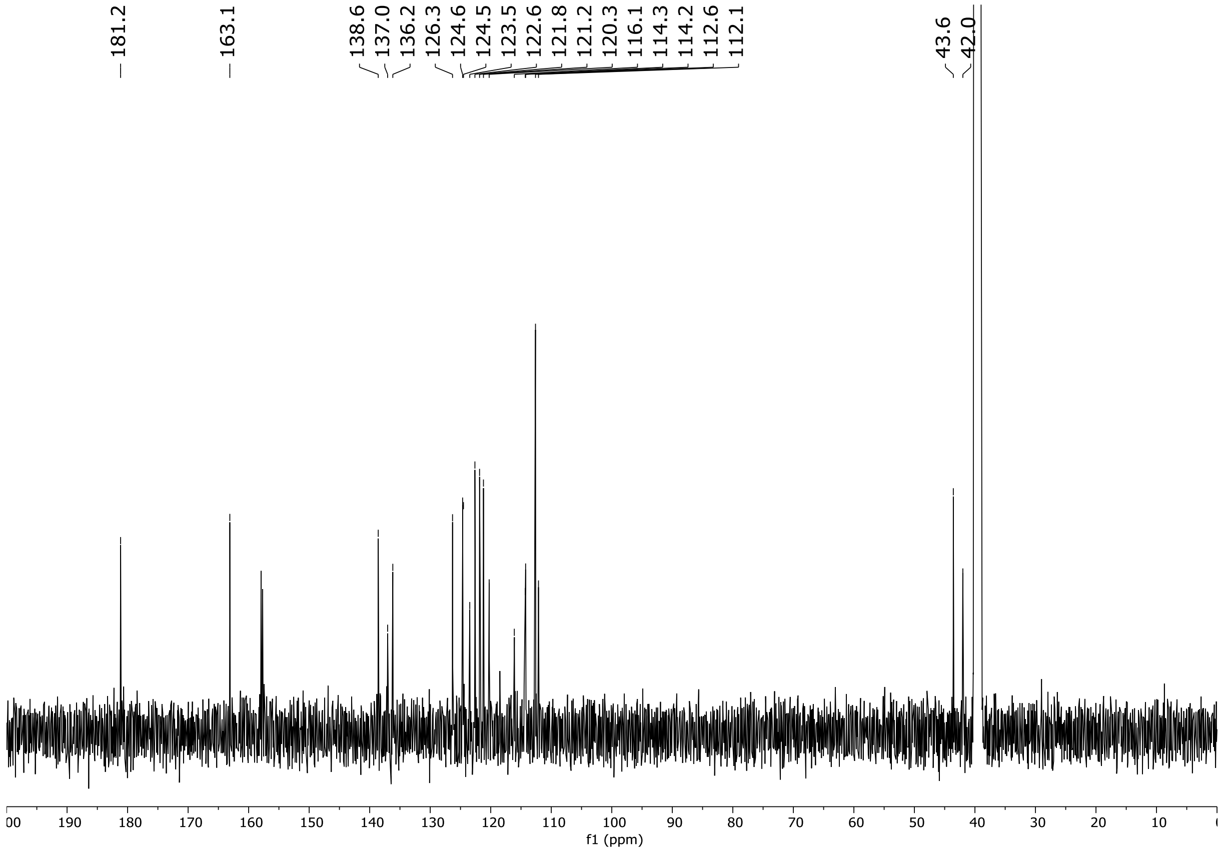

Figure S2. ^{13}C NMR spectrum of **1** at 125 MHz in DMSO-*d*6.

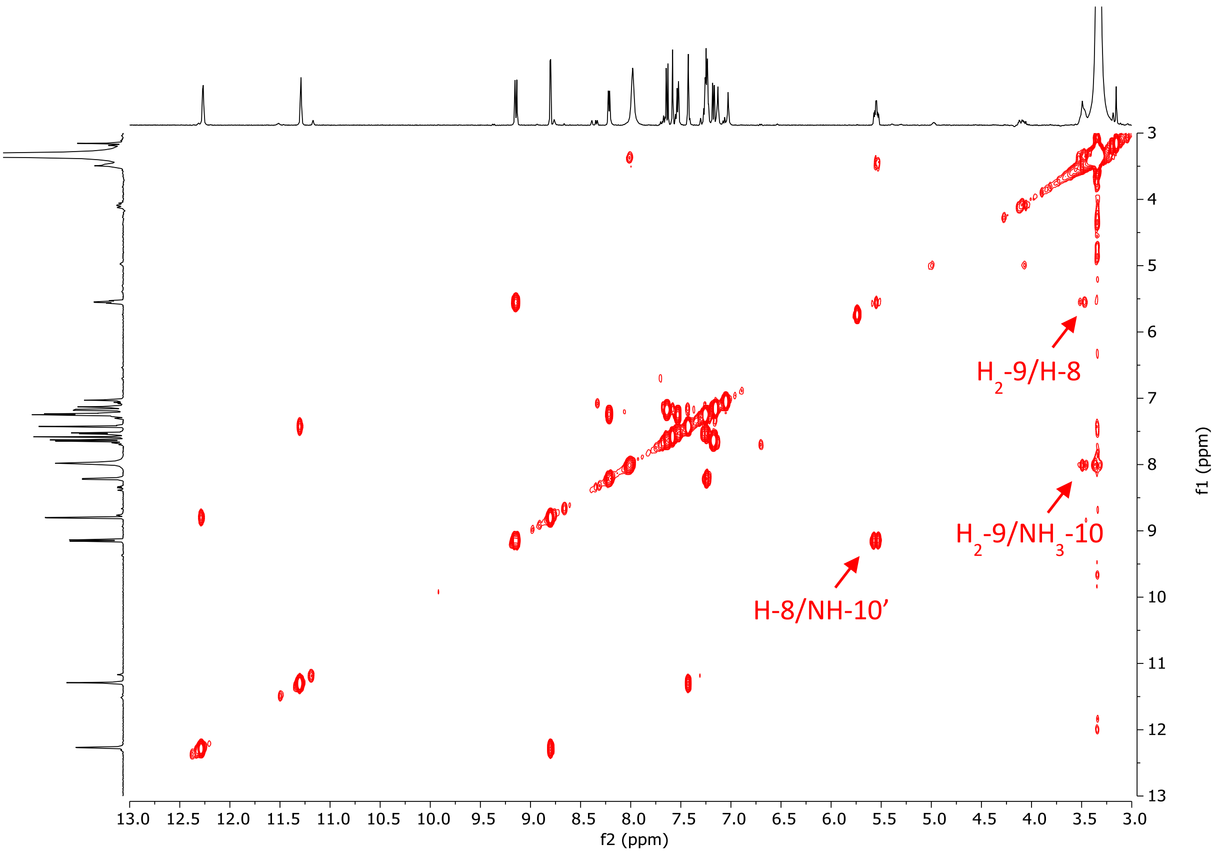

Figure S3. COSY NMR spectrum of **1** at 500 MHz in DMSO-*d*6.

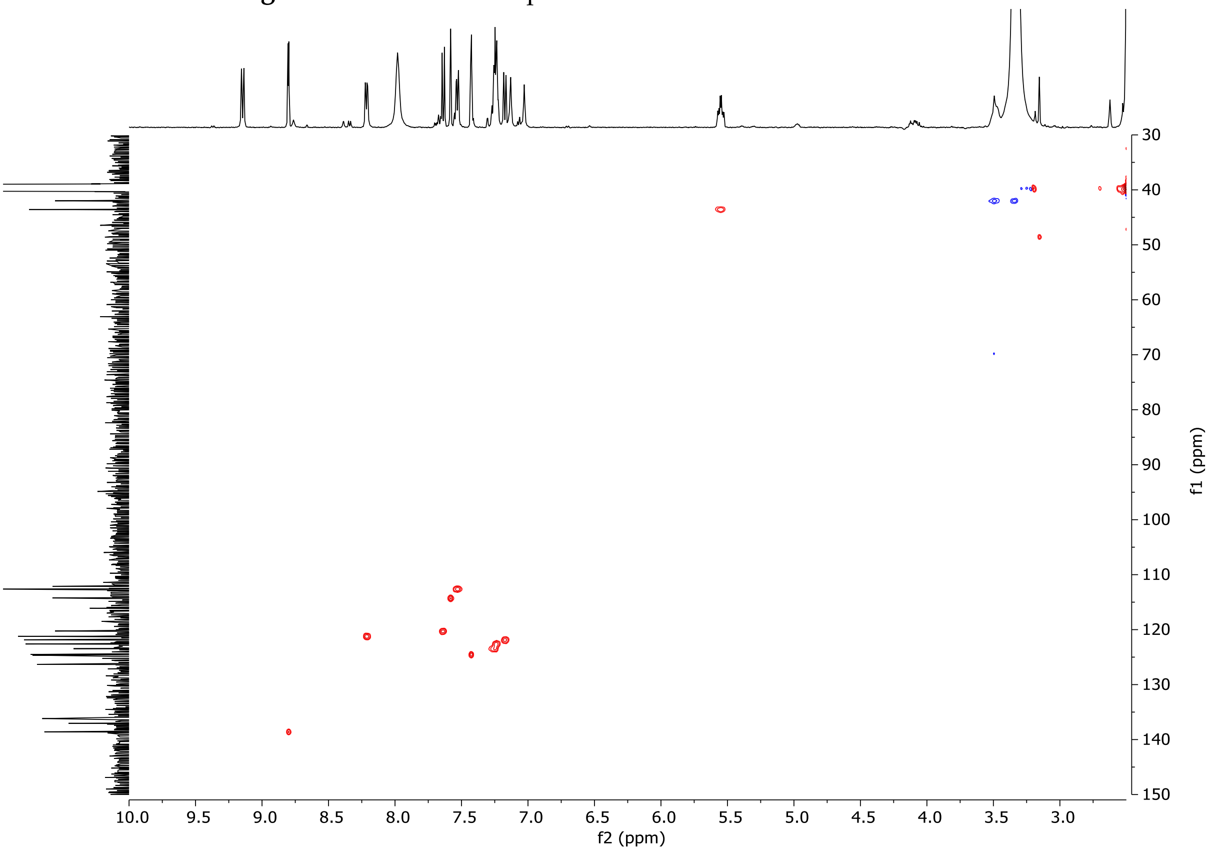

Figure S4. HSQC NMR spectrum of **1** at 500 MHz in DMSO-*d*6.

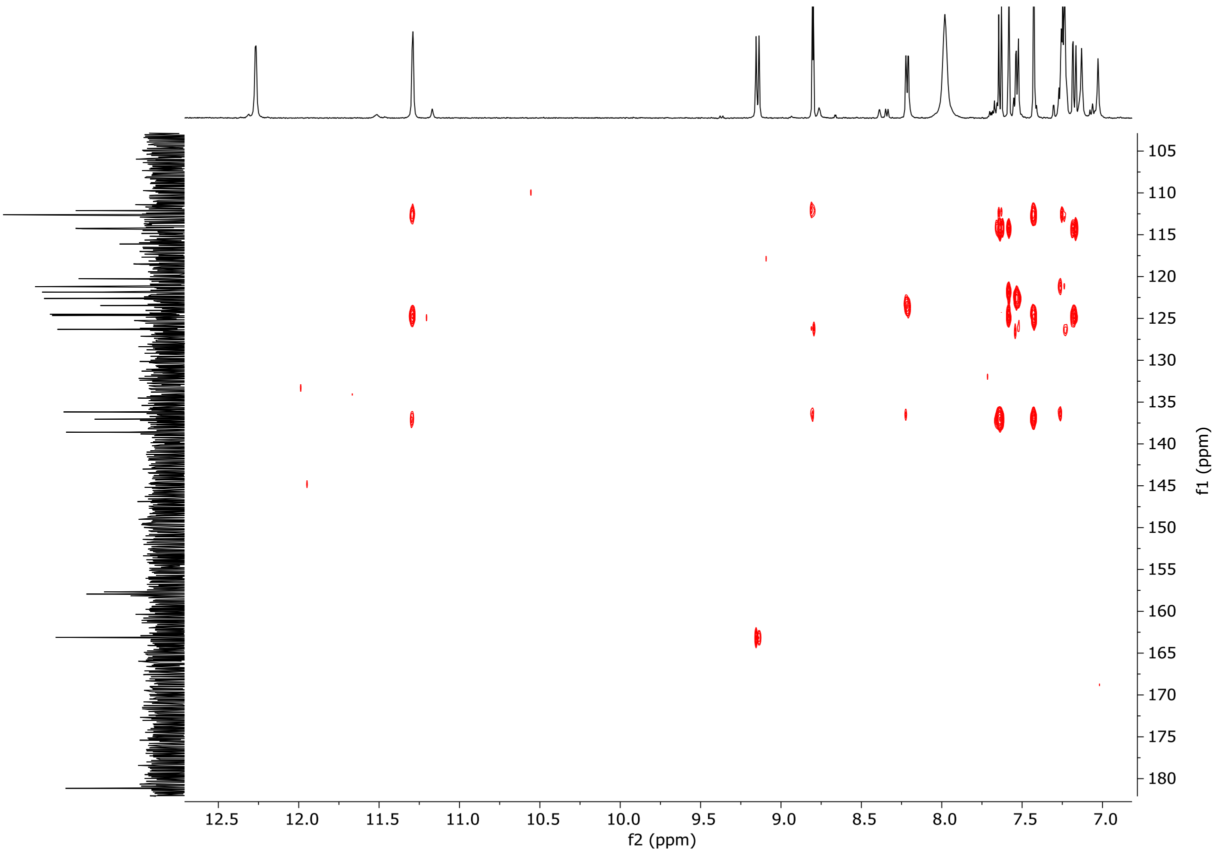

Figure S5. HMBC NMR spectrum of **1** at 600 MHz in DMSO-d_6.

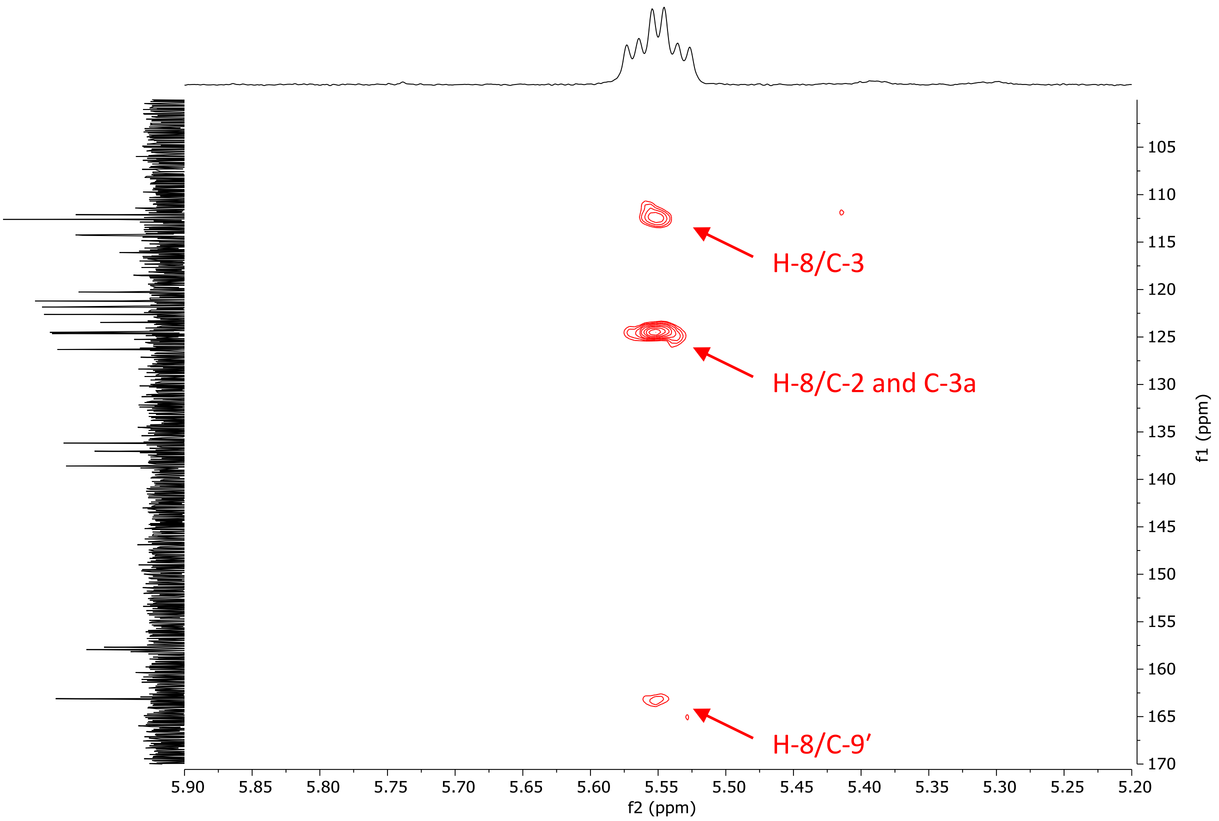

Figure S6. HMBC NMR spectrum of **1** at 600 MHz in DMSO-d_6.

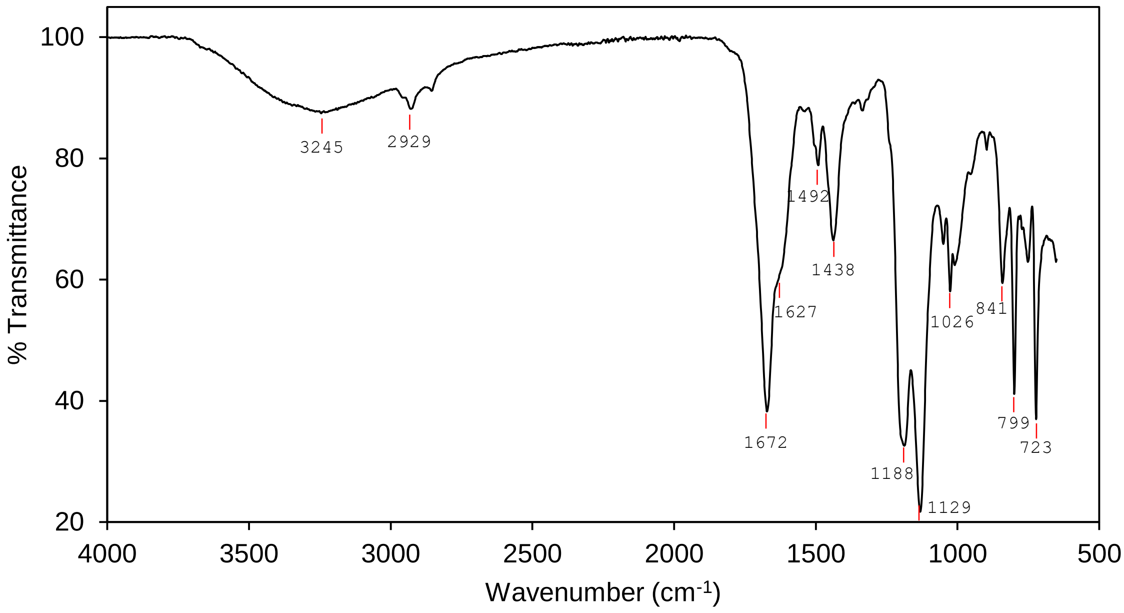

Figure S7. IR spectrum of **1**.

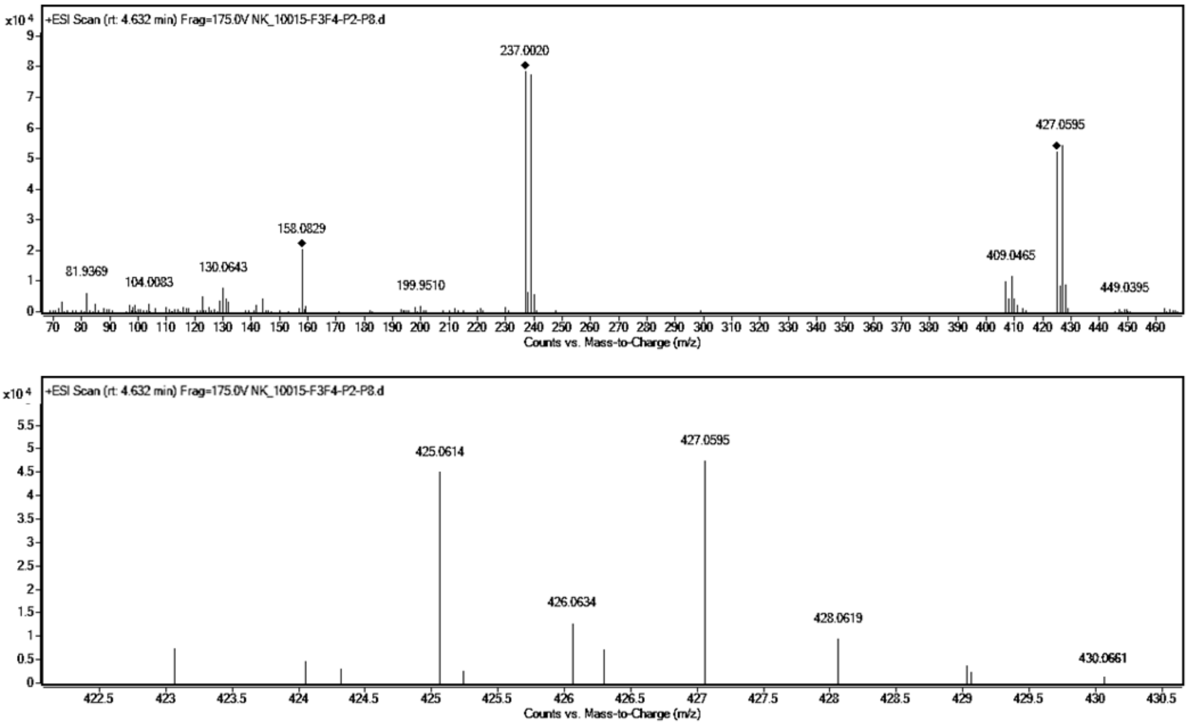

Figure S8. (+)ESIHRMS spectrum of **1**

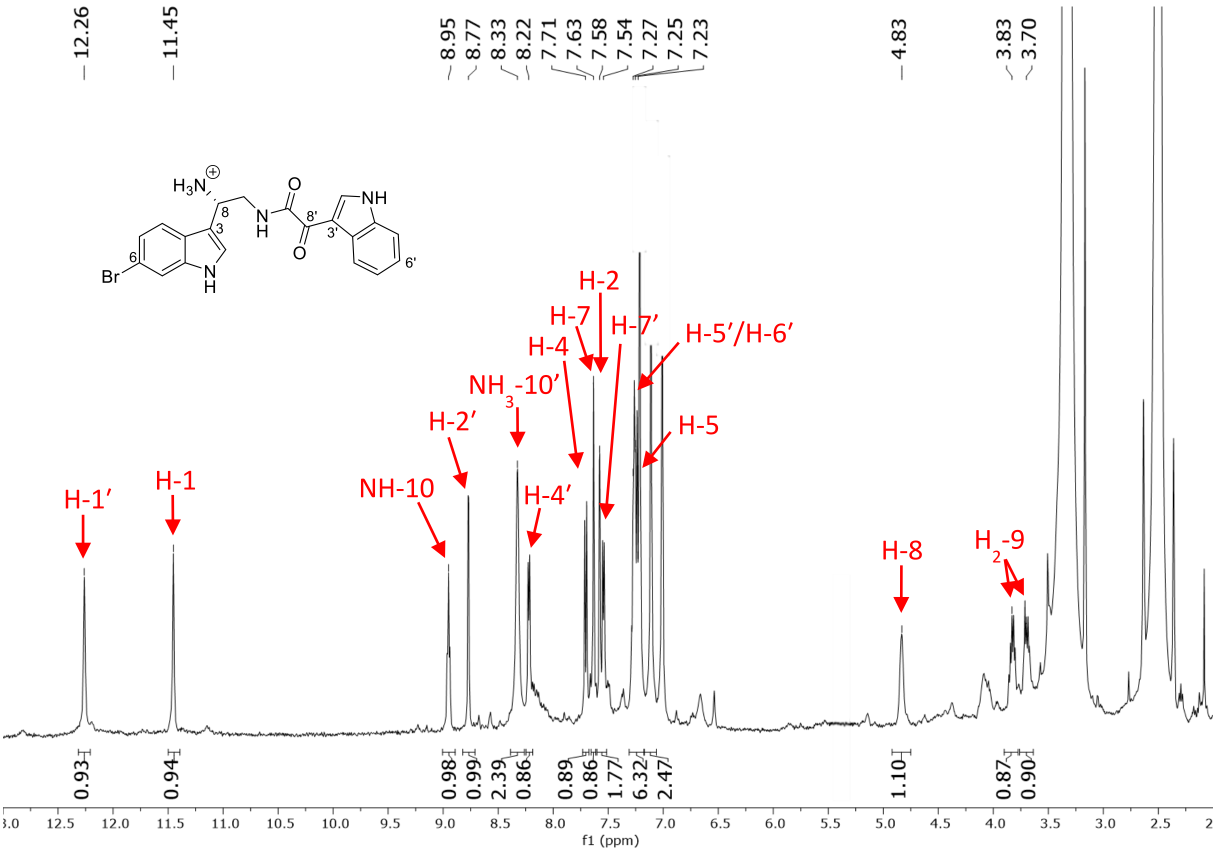

Figure S9. ^{1}H NMR spectrum of **2** at 500 MHz in DMSO-d_6.

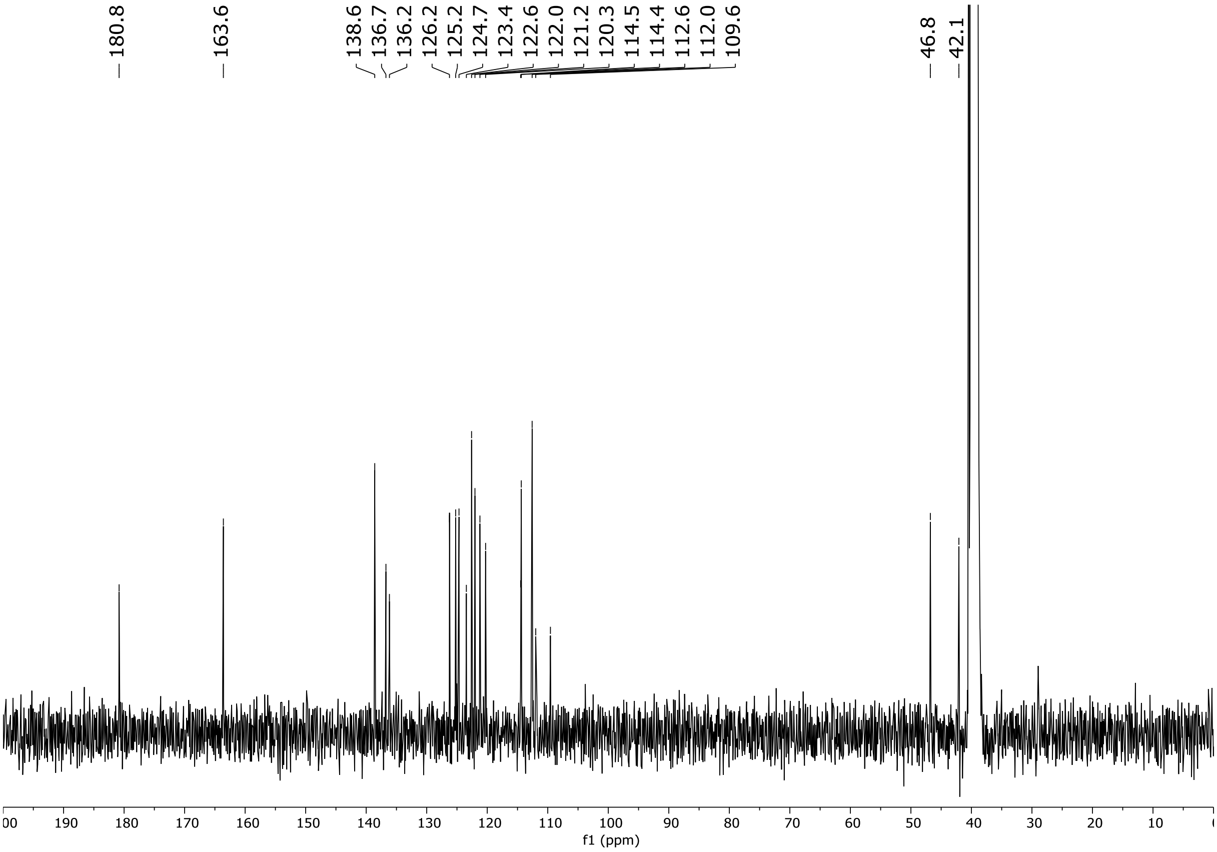

Figure S10. ^{13}C NMR spectrum of **2** at 125 MHz in DMSO-d_6.

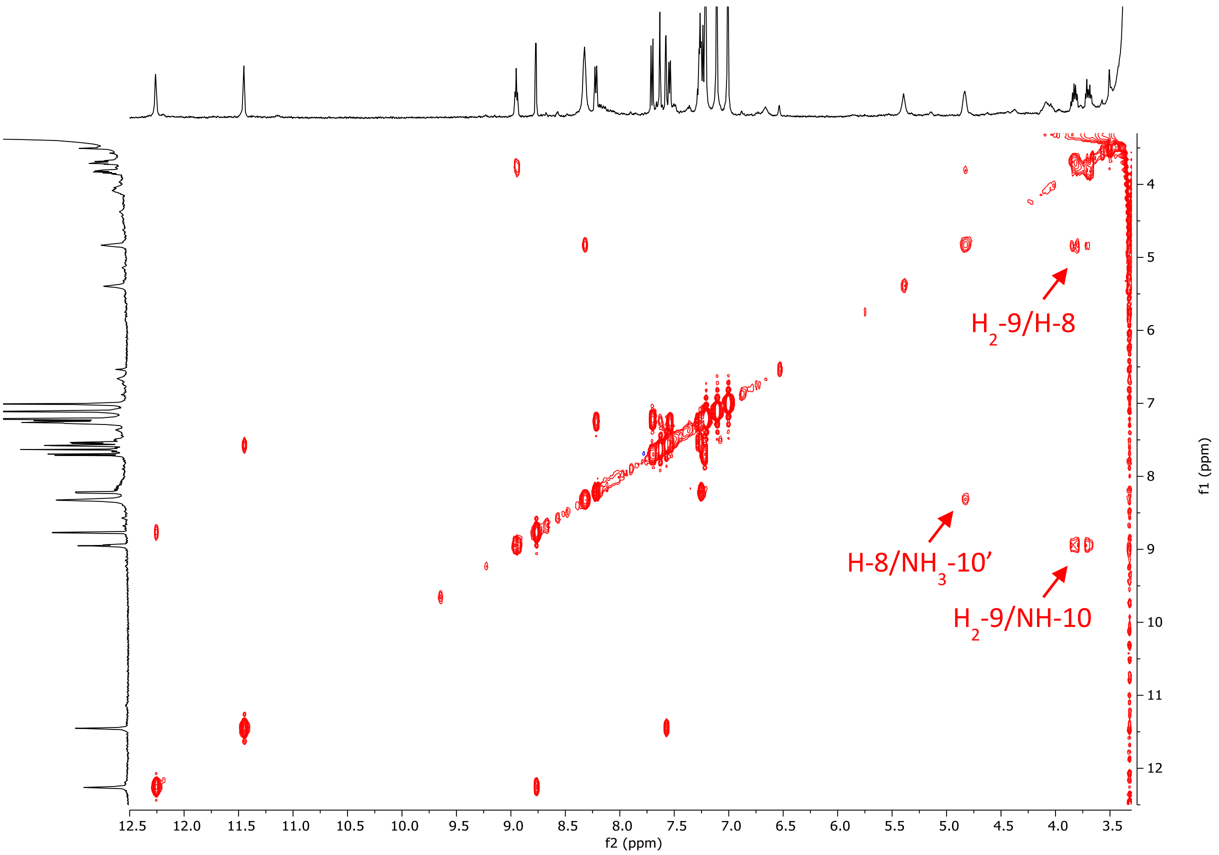

Figure S11. COSY NMR spectrum of **2** at 500 MHz in DMSO-*d*6.

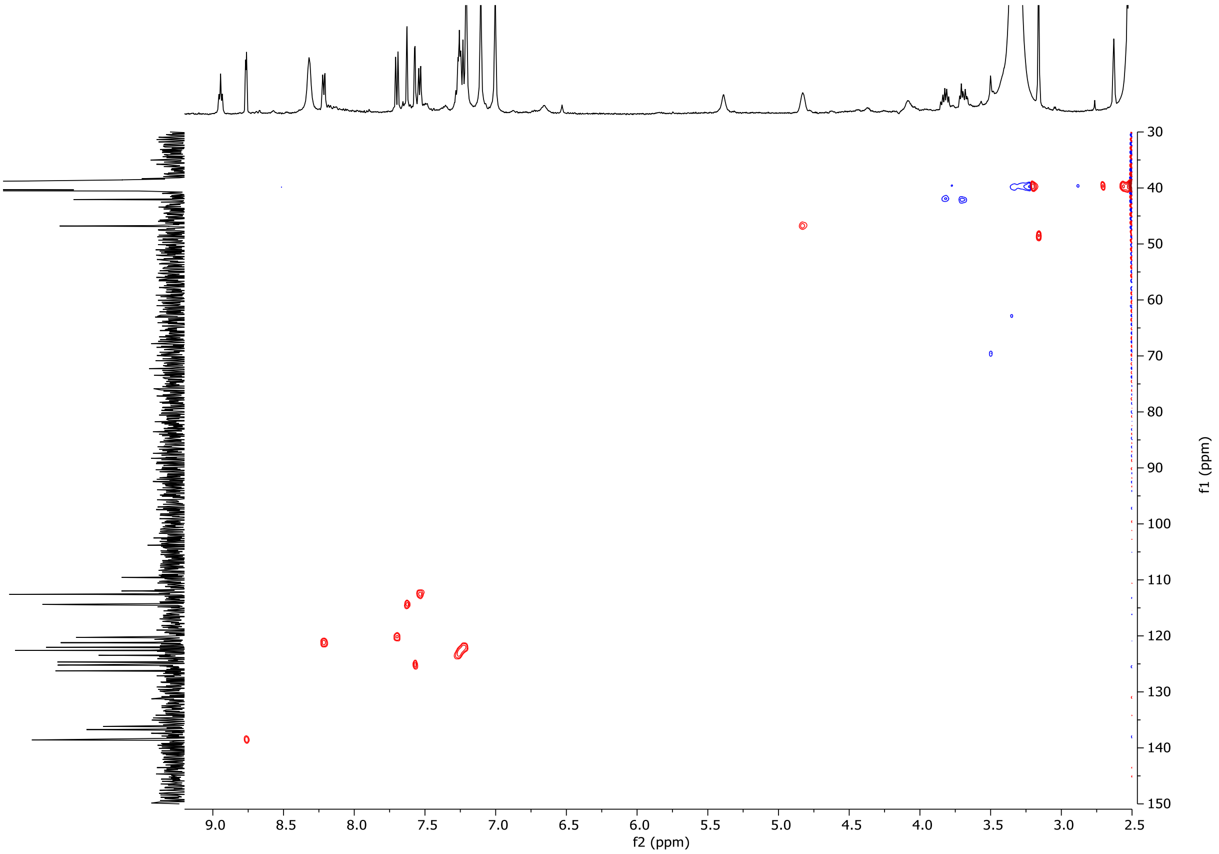

Figure S12. HSQC NMR spectrum of **2** at 500 MHz in DMSO-*d*6.

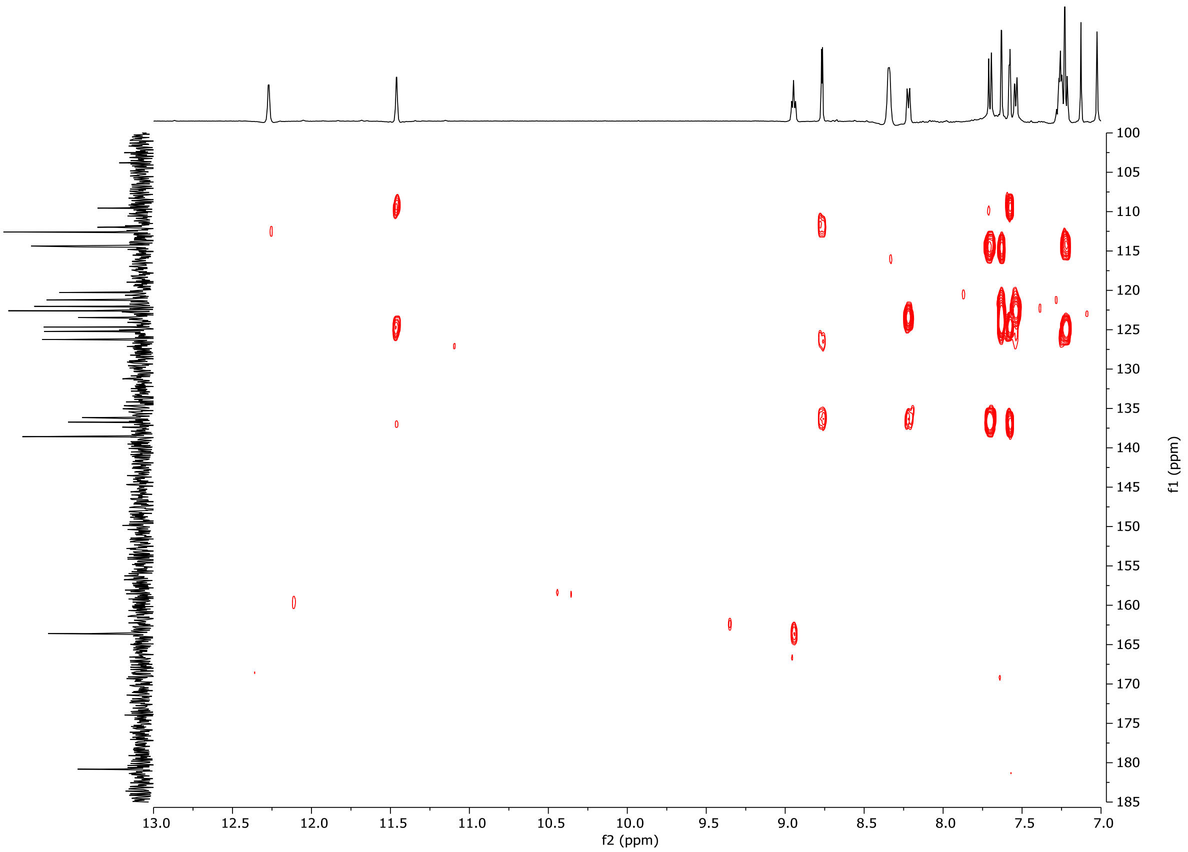

Figure S13. HMBC NMR spectrum of **2** at 500 MHz in DMSO-d_6.

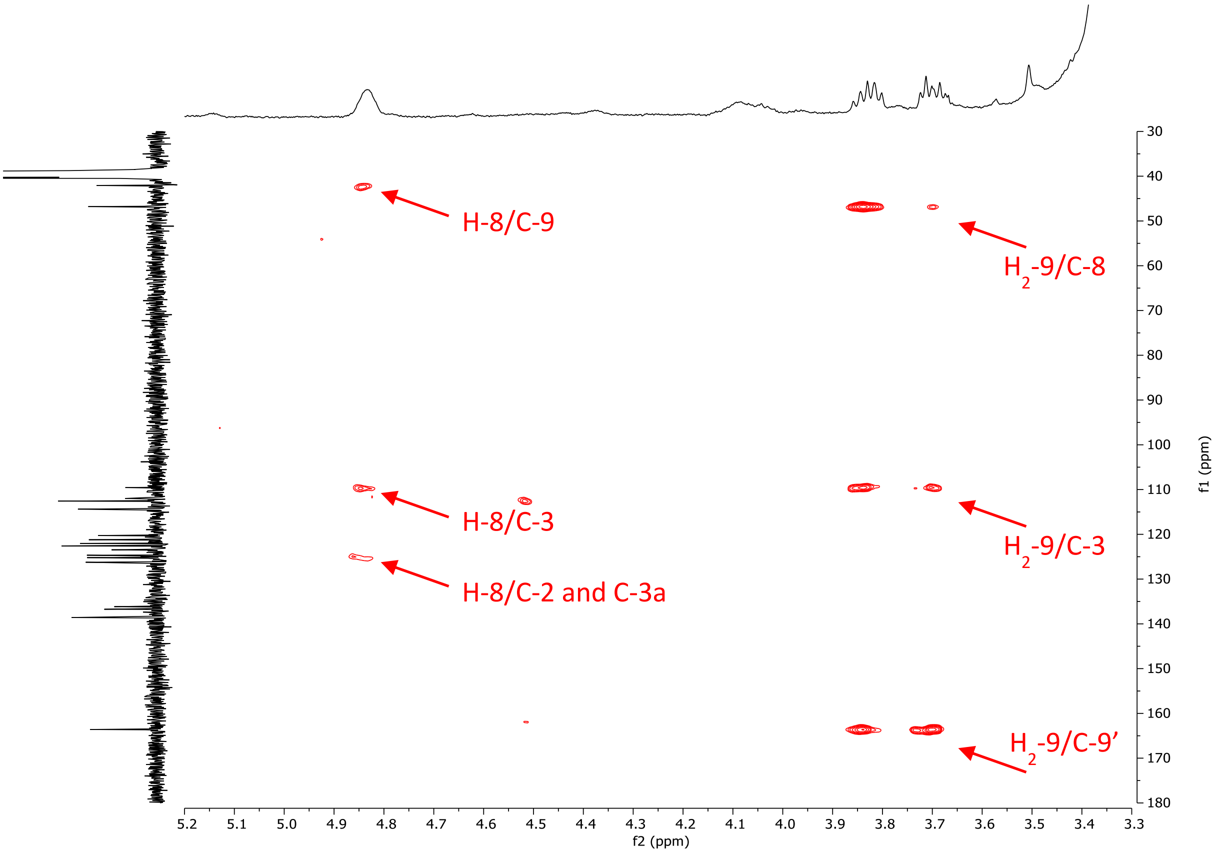

Figure S14. HMBC NMR spectrum of **2** at 500 MHz in DMSO-d_6.

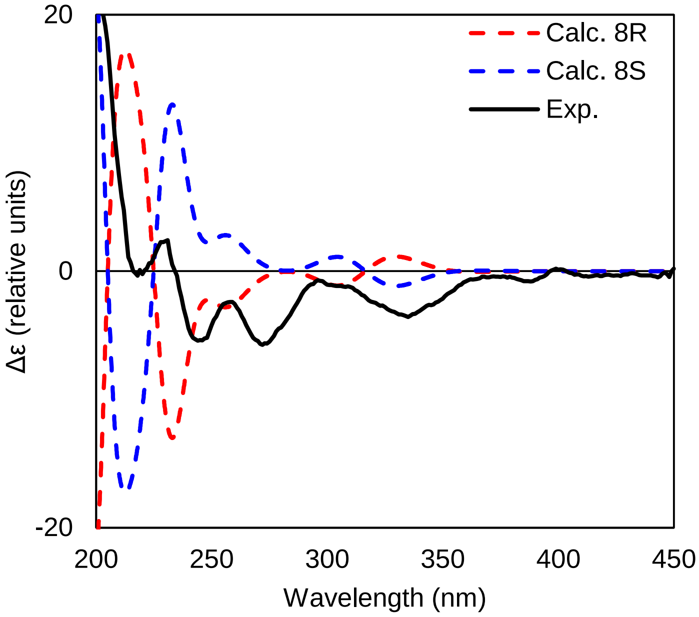

Figure S15. The comparison between the experimental and calculated ECD spectrum of **2**. The spectra were calculated using the B3LYP/6-311+G(2d,p)//B3LYP/6-311(2d,p) basis set/functional combination.

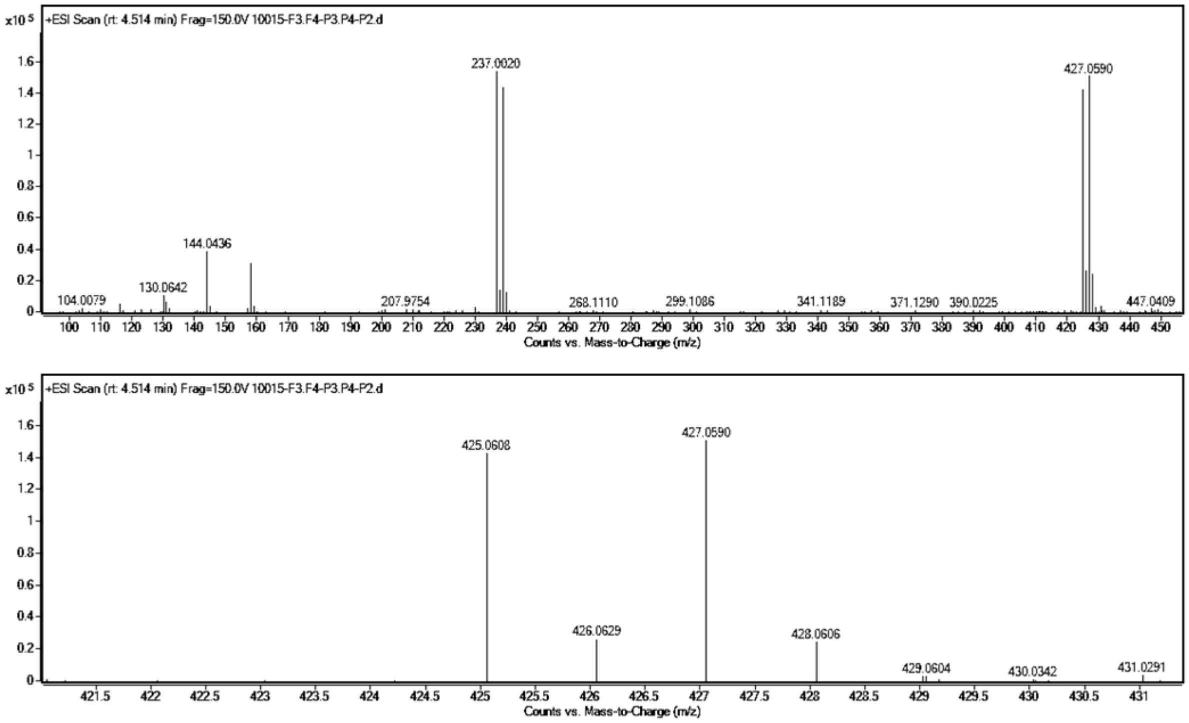

Figure S16. (+)ESIHRMS spectrum of **2**

Table S1. Cytotoxicity (μM) of compounds **1**-**6** against HeLa cells measured after 6 and 24 h incubation.

Compound	Cell Lysis IC_{50}	
	**6 h**	**24 h**
	(IC_{50} μM ± SD)	(IC_{50} μM ± SD)
Calcicamide A (**1**)	>200	>200
Calcicamide B (**2**)	165 ± 7	146 ± 13
trans-3,4-Dihydrohamacanthin A (**3**)	>200	>200
Bromodeoxytopsentin (**4**)	>200	125 ± 2
Bromotopsentin (**5**)	>200	123 ± 13
Spongotine A (**6**)	136 ± 5	115 ± 4

Table S2. Cytotoxicity of fractionated sponge extracts at 100 μg/mL against HeLa cells measured after 6 and 24 h incubation.

Sponge species	Fraction[a]	% Cell Lysis at 100 μg/mL	
		**6 h**	**24 h**
		(% ± SD)	(% ± SD)
	fwm	2±2	6±3
Axinella dissimilis	fm	2±1	8±4
	fmd	3±1	6±4
	fwm	3±1	6±3
Clathrina coriacea	fm	3±2	16±5
	fmd	3±2	9±7
	fwm	3±2	5±3
Clathria (Microciona)	fm	3±2	5±2
strepsitoxa	fmd	3±3	6±1
	fwm	4±2	5±2
Halichondria panicea	fm	2±2	3±1
	fmd	2±2	4±1
	fwm	2±2	6±2
Lophon hyndmani	fm	4±2	13±6
	fmd	4±2	9±5
	fwm	3±2	6±3
Mycale rotalis	fm	4±1	4±1
	fmd	4±3	5±2
	fwm	2±1	5±2
Polymastia boletiformis	fm	2±1	6±5
	fmd	2±1	3±2
	fwm	4±3	8±6
Polymastia penicillus	fm	8±3	23±6
	fmd	3±1	8±4
	fwm	**43±8** [b]	**61±6** [b]
Spongosorites calcicola	fm	15±6	17±2
	fmd	3±2	6±4
	fwm	3±2	6±4
Tethya sp	fm	2±1	4±1
	fmd	6±3	10±1

[a] SPE fractions of crude extracts: desalting: water elution; fwm : water:methanol (1 :1) elution; fm: methanol elution; fmd: methanol:dichloromethane (1 :1) elution. [b] Bold values indicate cytotoxicity 2 standard deviations from the mean cytotoxicity.
